# Modeling Warfare in Social Animals: A "Chemical" Approach

**DOI:** 10.1371/journal.pone.0111310

**Published:** 2014-11-04

**Authors:** Alisa Santarlasci, Gianluca Martelloni, Filippo Frizzi, Giacomo Santini, Franco Bagnoli

**Affiliations:** 1 Department of Information Engineering, University of Florence, Firenze, Italy; 2 Department Physics and Astronomy, University of Florence, Sesto Fiorentino (FI), Italy; 3 Center for the Study of Complex Dynamics (CSDC), University of Florence, Sesto Fiorentino (FI), Italy; 4 Department of Biology, University of Florence, Sesto Fiorentino (FI), Italy; 5 National Institute of Nuclear Physics (INFN), sez. Firenze, Italy; Instituto de Fisica Interdisciplinar y Sistemas Complejos IFISC (CSIC-UIB), Spain

## Abstract

We present here a general method for modelling the dynamics of battles among social animals. The proposed method exploits the procedures widely used to model chemical reactions, but still uncommon in behavioural studies. We applied this methodology to the interpretation of experimental observations of battles between two species of ants (*Lasius neglectus* and *Lasius paralienus*), but this scheme may have a wider applicability and can be extended to other species as well. We performed two types of experiment labelled as *interaction* and *mortality*. The *interaction* experiments are designed to obtain information on the combat dynamics and lasted one hour. The *mortality* ones provide information on the casualty rates of the two species and lasted five hours. We modelled the interactions among ants using a chemical model which considers the single ant individuals and fighting groups analogously to atoms and molecules. The mean-field behaviour of the model is described by a set of non-linear differential equations. We also performed stochastic simulations of the corresponding agent-based model by means of the Gillespie event-driven integration scheme. By fitting the stochastic trajectories with the deterministic model, we obtained the probability distribution of the reaction parameters. The main result that we obtained is a dominance phase diagram, that gives the average trajectory of a generic battle, for an arbitrary number of opponents. This phase diagram was validated with some extra experiments. With respect to other war models (*e.g.*, Lanchester's ones), our chemical model considers all phases of the battle and not only casualties. This allows a more detailed description of the battle (with a larger number of parameters), allowing the development of more sophisticated models (*e.g.*, spatial ones), with the goal of distinguishing collective effects from the strategic ones.

## Introduction

Many social animals fight in groups in the context of intra- or inter-specific competition [1], [2]. Fighting in a group differs considerably from fighting individually, since the outcome of a combat is not simply dictated by the ability of each individual (usually known as *individual fighting ability* or *resource holding potential*). We can distinguish between collective effects (due to the number of participants) and strategic ones (changes of behavior for obtaining a specific goal).

Both the size of fighting groups and the ability to coordinate within-group agonistic behaviors may play a role (see *e.g.* Refs. [3]–[5]). Broadly speaking, two main type of battles may be recognized. At one extreme, a battle is formed by a series of individual duels and members of the larger group remain disengaged at the side of the battle until an opponent become available. At the other extreme, members of the larger group may cooperate in attacking isolated members of the smaller one. In the latter case, the group size may have a disproportionate importance on the final outcome of the battles whereas in the former the ability of each individual to fight may be the key factor [2]. A strategic approach would imply for instance the choice of the more advantageous situations giving the available resources.

An interesting mathematical framework for the analysis of the attrition rates during group battles is represented by the so-called Lanchester laws. Lanchester [6], [7] formulated two models, known as the "linear" and the "square" laws, to describe aerial combats during the first world war. Since their early formulation, these models (and their further developments) have been extensively used to model battle outcomes in the sphere of human conflict (see *e.g.* Refs. [8]–[11]). Lanchester theory has been applied to animal contests too, with varying degrees of success (see *e.g.* Refs. [4], [5], [12]–[16]). The great appeal of these models lies in their simplicity, since they allow to estimate the attrition rates of two opposing armies solely as a function of their number and fighting ability of individuals within each group. One critical point when willing to apply these laws to animal warfare is that they rely on a number of restrictive assumption, which may not apply in animal combats (reviewed in Ref [2]), such as use of long-range weapons which are common in human warfare but rare in animal combats, where a close contact between the opponents is required. As a consequence of their limited biological realism, predictions obtained from these models have to be considered carefully, although they may provide interesting mean-field approximations to the dynamics of aggressive encounters. The possibility to have a sound and coherent conceptual framework to investigate animal fights may have important consequences. On one hand, given a detailed model of an animal war, one can investigate the discrepancies between the model and the observed behaviour, hoping to be able to distinguish between the collective effects of the interacting elements (included in the model) from other effects due, for instance, to cognitive strategies not included into the model. On the other hand, predicting the outcome of combat may prove to be a fundamental tool in applied control science when willing to predict the spread of invasive species or the expansion range of some species following climate changes, both of which are well recognized among the most serious threats to world biodiversity [17].

A promising approach to the dynamical modelling of ecological systems is represented by the "chemical' approach', where all the interactions among members of the system are described following the formalism used to represent chemical reactions [18], disregarding complex cognitive strategies. Despite their widespread use to model chemical reactions and their recent introduction into ecology (see *e.g.* [19]), no attempt have been made, to our knowledge, to applied these approach in behavioural studies. In the specific case of aggressive encounters, all the possible combinations of combatants (isolated individuals of the two bands, groups of gripping fighters which form during the course of the battle) may be equated to the molecules ("chemical species") involved in a chemical reaction. For instance, one individual belonging to species 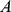 (chemical species 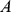) can stick to an enemy of species 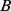 (chemical species 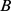) to form a group, considered as an instance of a chemical species called 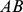, according to the formula:
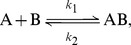
where 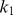 and 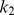 are the rate constants, which express the probability that this reversible reaction may occur per unit time.

One of the great advantages of this approach is that all possible interactions can be outlined using a simple but biologically meaningful formalism, which provides a "microscopic", individual-level description of the system, to be contrasted to the "macroscopic", population-level description provided for instance by the classical Lanchester models. Once all the relevant reactions are identified, these can be converted into a system of differential equations to get a mean field description of the system, or used to perform stochastic simulations (*e.g.*, using the Gillespie algorithm [20]), to explore and quantify the effect of variability on the behaviour of the system under investigation.

Our approach was developed taking as reference the interactions between two ant species *Lasius neglectus* (an invasive species) and *Lasius paralienus* (an autochthonous species), for which multi-party combats were staged. Once all the possible reactions were identified, a system of differential non-linear equations that allows a mean-field description of the system was deduced and fitted to observed data. Stochastic simulations were also obtained solving the model using the Gillespie algorithm and these were compared to the mean-field description in order to estimate the errors of the experimental rate coefficients.

The main goal of this approach is that of obtaining reliable rate coefficients that can be further interpreted in terms of a smaller number of parameters like aggressiveness, yieldingness, strength, etc. and that could give insights into the strategies used by opponents.

## Materials and Methods

### Experimental setup: ant sampling and types of experiments

Ants of the two studied species (*Lasius paralienus* and *Lasius neglectus*) were collected during July/August 2013 in Prato (Northern Tuscany, Italy, 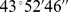
N, 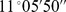
E). According to Italian laws no specific permissions were required for collecting ants and performing the experiments. The study did not involve endangered or protected species and sampling was not carried out in a protected area. *L. paralienus* [21] is an endemic species, widespread in central Europe and part of the Mediterranean, forming medium to small colonies and generally showing little competitive ability. *Lasius neglectus* is an *invasive species*, which recently spread its range throughout Europe, causing serious concerns for its impact on native species [22]. This species is highly dominant, aggressive and form large supercolonies which extend over wide areas. Both species are monomorphic with reduced intraspecific differences in the size of ants. Congeneric species were chosen to reduce confounding due to the adoption of different combat modes. Ants were captured during the morning and stored in 50 ml test tubes, with water available, to acclimate to laboratory conditions for one hour (temperature 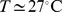) before being used in an experiment. Two different types of experiment were carried out. The aim of the first type, hereafter referred to as *interaction experiments*, was to obtain information on combat dynamics. In particular we monitored how ants interacted and formed fighting groups, *i.e.* group composed by two or more specimens, which remain in close contact for at least 20 seconds, biting or spraying venom. To avoid confounding effects due to fatigue or changes in individual motivation to fight, the duration of these experiments was maintained short (1 hour). After acclimation, 10 specimens of the two species were simultaneously dropped within a neutral arena, consisting in a 10 cm Petri dish with Fluon coated walls and their behaviour continuously recorded for the following hour using a digital camera.

**Figure 1:**
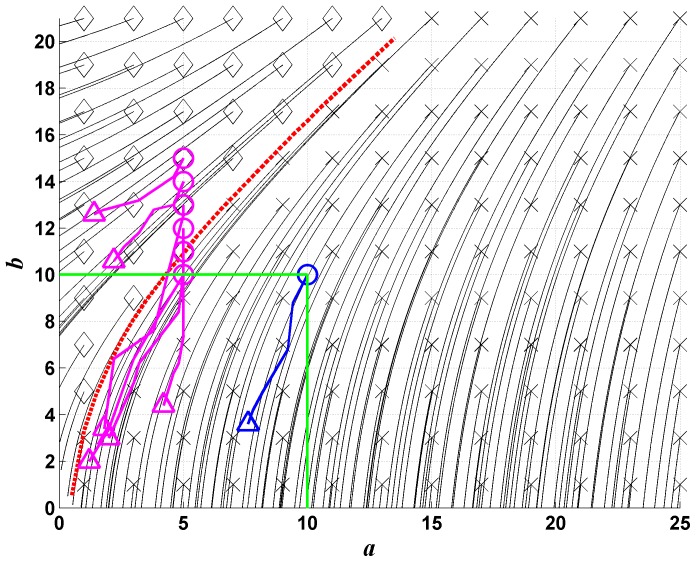
Supremacy phase diagram. Evolution of the trajectories of the deterministic model starting from different initial conditions, projected on the plane determined by the total number of A individuals (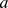) and the total number of B individuals (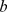). Initial conditions indicated by diamonds lead to the supremacy of 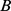
's (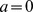), while the x-marks lead to the supremacy of the species 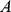 (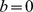). The red dashed line is the separatix between the two phases, where both species die; the green square marks the region where fitting has been performed and where non-linear effects are most effective. The magenta and the blue lines indicate experimental trajectories, each one given by an average over 5 experiments.

We choose this number as a compromise between the observability of the individual insects and a numerosity sufficiently high not to affect the formation of fighting groups. Preliminary observations showed that the largest groups are formed by four insects, so that with 10 opponents of each species there is room for several groups also in the presence of casualties. This choice is confirmed a posteriori by the fact that the non-linear effects are much more pronounced for numerosity of each species less than 10 (see Fig. 1).

**Figure 2:**
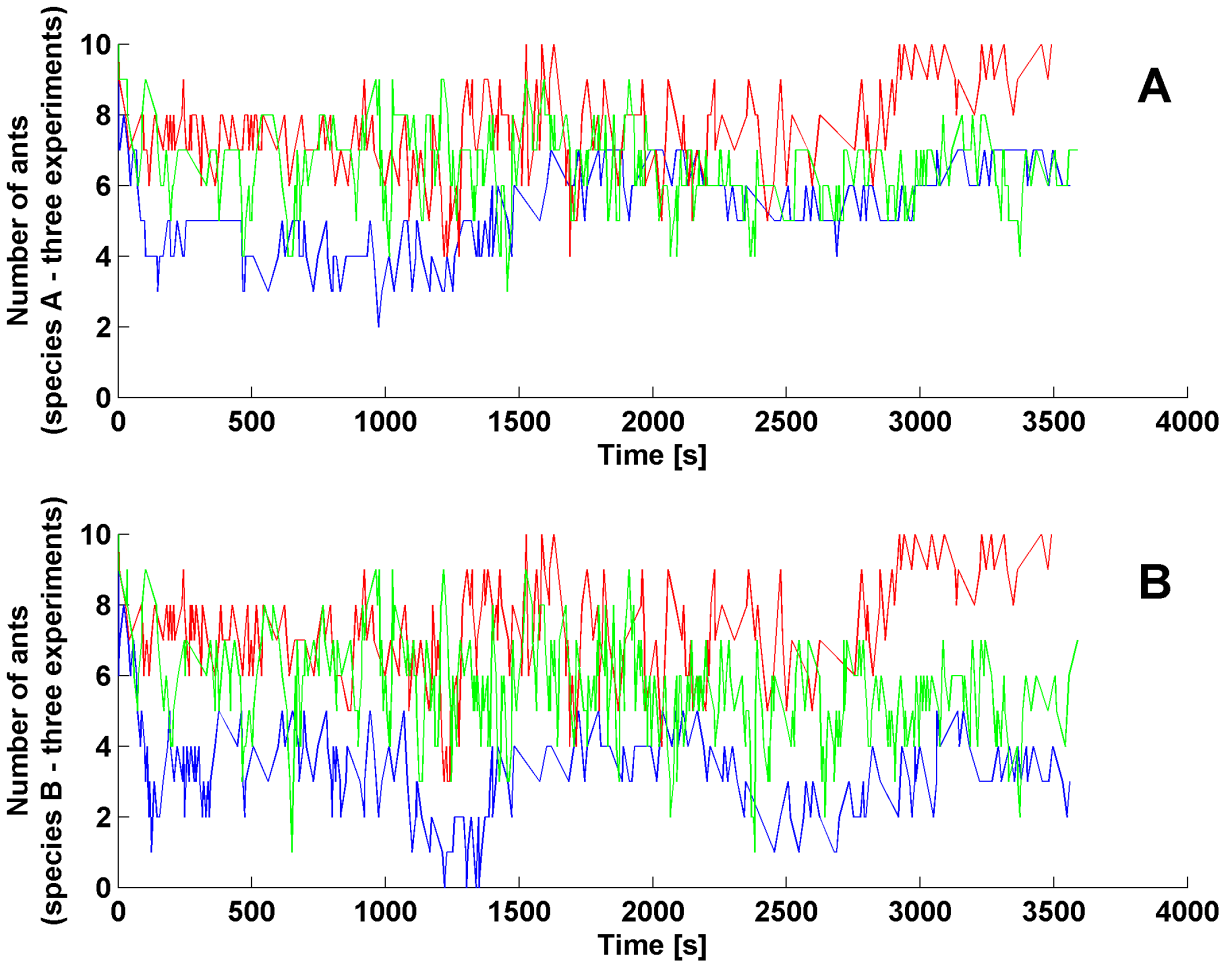
Experimental data. Abundance of 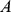 (subfigure (a)) and 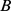 (subfigure (b)) species from three different experiments.

A total of 20 replicate experiments were performed. The videos were then analysed, detecting and recording the appearance and variation of all the fighting groups of interacting ants. The sampling is done per event, *i.e.*, we recorded the time when an event (for instance the formation of a group) occurred. An example of the resulting data is shown in Table 1 while Fig. 2 shows three examples of experimental time series. All data and videos are available from the corresponding authors upon request.

**Table 1:** An example of experimental data.

Event time 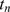	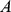 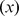	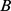 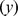	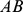 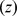	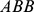 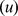	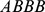 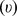
714	6	5	3	1	0
741	5	3	3	2	0
751	5	2	3	1	1
778	4	1	4	1	1
781	4	2	5	0	1
783	4	3	5	1	0
797	5	4	4	1	0
801	5	2	2	3	0
803	5	3	3	2	0
805	5	4	4	1	0
821	5	3	3	2	0
824	5	3	4	1	0
830	6	5	3	1	0
832	5	4	4	1	0
846	5	3	3	2	0
850	6	5	3	1	0
858	5	3	3	2	0

The first column shows the occurrence time (in seconds) of a given reaction (event). The other columns (2–6) report the total number of each chemical species (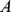, 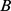, 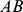, 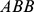 and 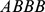.

The two species exhibit a slightly different behaviour: in most cases *L. neglectus* is the first aggressor and cooperation among nestmates against opponent was observed but also suffered the greater mortality. *L. paralienus*, on the contrary, is slightly greater in size, stronger in individual duels but less aggressive than *L. neglectus*. Due to the behaviour of the two species and denoting *L. paralienus* as 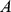 and *L. neglectus* as 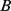, the following groups may form: 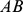, 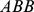 and 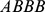. No observation of more than one *L. paralienus* (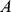) attaching a single *L. neglectus* (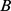) was done and hence other groups (es. 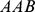) were not considered in subsequent analysis. The combination 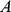, 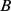, 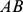, 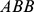 and 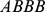 are referred to as "chemical species" or "molecules", following the terminology used in chemical modeling [18].

The second type of experiments, hereafter referred to as *mortality experiments* aimed to estimate the attrition rates of the two species for a different initial size of the two groups. Seven different sets of starting numbers were used, as shown in Table 2. The two groups of ants were simultaneously dropped within a 10 cm Petri dish with Fluon coated walls and the number of dead ants was counted each at intervals of one hour there were individuals able to fight (alive and not injured) or up to a maximum of 5 hours.

**Table 2:** Means of 5 experiments for 7 different initial conditions for the duration of 5 hour (Set 1, Set 2, etc.); *L. paralienus* (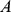), *L. neglectus* (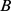).

Observations (time [s])	Set 1 (survivors)		Set 2 (survivors)		Set 3 (survivors)		Set 4 (survivors)		Set 5 (survivors)		Set 6 (survivors)		Set 7 (survivors)	
	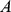	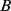	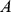	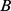	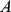	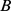	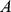	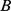	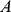	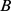	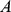	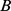	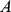	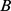
0	10	10	5	10	5	11	5	12	5	13	5	14	5	15
3600	9.4	8.8	5	9.4	5	10	5	10.2	5	12.6	4.6	13	4.6	14.2
7200	9.2	6.8	5	7.4	4.2	8.6	4.8	8.4	4.4	10.2	3.8	12.8	3.2	13.2
10800	8.2	5	4.86	.2	3	6.4	3.2	6.2	3.8	7.6	3.2	11.8	1.8	12.8
14400	7.8	4.2	4.6	5.8	2	3.4	3	5.4	2.2	6.4	2.6	11.2	1.4	12.6
18000	7.6	3.6	4.2	4.4	1.2	2	2	3	1.8	3.4	2.2	10.6	1.4	12.6

### Chemical model

The model consists in a collection of chemical equations, which encode for the interactions among individual entities. The first reaction happens when an individual of *L. neglectus* (chemical species 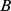) establishes a strong tie in fight with an individual of *L. paralienus* (chemical species 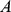) to form a new group or chemical species 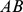,
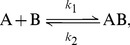
(1)where 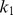 and 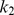 are the reaction constants of the direct and reverse reactions, respectively.

The outcome of a duel can lead to the death of 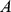,
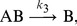
(2)or to the death of B,
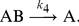
(3)


Once a group 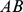 is formed, a second 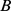 can participate in the fight. We describe the appearance of a group 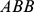 by means of the reversible reaction
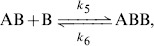
(4) since in the experiments we also observed cases in which a *B* detaches from the group.

In a fighting group 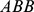, an 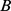 can die, giving the irreversible reaction
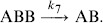
(5)


Then we add the possibility, as observed in the experiments, that also an *A* ant dies as consequence of a fighting with two 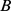 ants. In this case the group 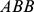 dissolves,
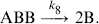
(6)


Another recurring possibility is that a group 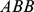 dissolves without any death but also the reverse reaction may occur, *i.e.* that two 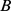 attack an 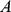:
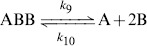
(7)


Attack by two 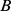 can be considered as simultaneous since it occur in a very short interval with respect to the observation time.

Observations also show the sticking of three 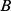
's with an 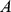, as described in the following reversible reaction, in which it there is also the possibility that a 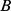 abandons the group,
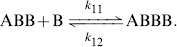
(8)


An individual 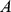 can die as a consequence of the fighting with three 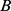
's and the group dissolves, *i.e.*,
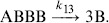
(9)


Finally another possibility is the detachment of two 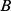
's from the group. Considering also the opposite reaction, we have
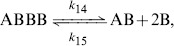
(10)where it is assumed that the two 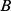
's attach to the group 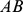 separately, but in a very short interval compared to the sampling time.

### Mean-field approximation

As usual in chemical studies, we may exploit the hypothesis of decorrelation and random movement of particles (ants), in order to obtain the *average* behaviour of the model, that using the physics terminology can be denoted the *mean-field* approximation.

Let us denote by 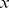 the number of 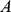 individuals, 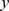 is the amount of 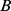
's, 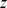 that of groups 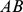, 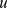 corresponds to groups 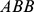 and finally 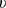 represent the number of groups 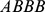. The above chemical reactions can be translated into a set of non-linear ordinary differential equations, describing the change in the numerosity of the different chemical species with respect to time:
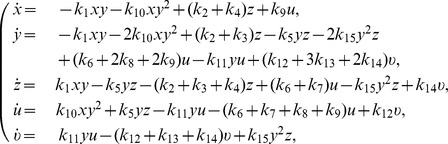
(11)where the dot denotes the derivative of each variable with respect to time.

It is possible to derive the trajectories of this system using standard ODE solvers (see File S1 for details).

The mean-field approximation does not exhibit fixed points, *i.e.*, the stationary solution (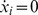) always depends on the initial conditions and parameter values. The stationary condition is either 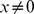 and 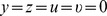 or 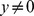 and 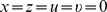, which can be denoted as absorbing states for the dynamics. In practice, this means that one of the two groups of opponents (biological species) goes extinct, while the other survives. Examples of predictions obtained from this model are shown in Figs. S1–S5. A fuller analysis of the behaviour of the deterministic model and an illustration of non-existence of fixed points is reported in the File S1.

### Stochastic approximation

To incorporate the consequences of finite-size fluctuations, due to the small size of the studied system (10 ants *vs.* 10 ants), we implemented a stochastic version of the model using the Gillespie's direct method [20], that again neglects spatial correlations. Given the limited spatial extension of the battlefield (Petri dish) and the limited time resolution (20s) we may in fact still assume that encounters between opponents occur randomly and use an event-driven approach, such as that used in chemistry to model "well-stirred" chemical reactions. Details of the implementation of the Gillespie method are given in File S1.

The methodology we adopted to compare the deterministic and the stochastic version of the model is the following. Firstly, we fit the deterministic model to the average experimental time-series to obtain an estimate of the 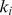 reaction constants. Secondly, we run 100 times the stochastic model using the above parameter values, and re-analysed the simulated time series as done with the experimental one obtaining a distribution of parameter values, that can be used to estimate the variance (and thus the presumed error) of the experimental parameters. A schematic representation of this procedure is shown in Fig. S6.

The parameter estimation was achieved using an heuristic optimization procedure, called the Simplex Flexible Algorithm (SFA) [23], based on the algorithm of Nelder and Mead [24]. Let us denote with 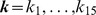 the set of reaction parameters, with 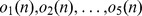, the observed experimental quantities sampled at times 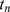, 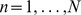 (see Table 1) and with 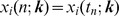, 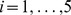 the corresponding variables obtained by integrating the differential system Eq.(11). The error function 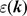 to be minimized is
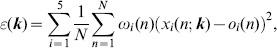
(12)where 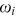 is a weight. Three different types of weight were tested: *i*) no weight, *ii*) the observed values 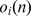 and *iii*) the absolute value of the derivative of the observed values 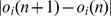. The lowest error 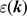 was obtained using the observed values as weight. The same approach was used for both the *interaction* and the *mortality* experiments. In the first case, observed and predicted values were represented by the abundances of the different chemical species, while in the second by the number of dead ants. In the latter case, only the 10 *vs* 10 ants experiments were used to fit the model while all other data were used to test the model predictions.

## Results

### Interaction experiments

The estimated values of the coefficients 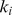 of the 15 reaction constants of the deterministic model are reported in Table 3. In the column labelled **ExpT** we list the value of parameters computed fitting the model with a time-average of all experimental data.

**Table 3:** Comparison among estimated reaction constants (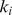), from all sets of data, averages from Gillespie simulations and experimental averages over single experiment.

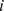	Reaction	*ExpT*	*Gill*	*Exp*
1	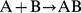	1.0063e-003	1.3178e-003	1.5583e-003
2	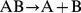	1.3130e-002	1.6753e-002	2.7332e-002
3	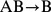	3.5892e-007	4.1913e-007	7.0946e-007
4	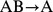	5.8290e-005	4.3790e-005	1.3909e-005
5	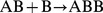	8.8645e-004	1.1091e-006	3.3791e-005
6	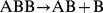	2.1387e-002	3.9210e-002	5.8170e-002
7	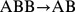	6.1787e-005	5.9673e-005	8.0680e-005
8	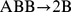	1.2312e-005	1.1618e-005	1.1173e-005
9	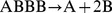	1.0528e-003	1.6991e-003	1.6387e-003
10	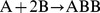	2.1974e-005	1.1079e-006	3.9706e-006
11	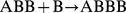	5.8482e-004	7.5142e-004	9.7736e-004
12	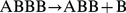	1.0246e-001	5.7494e-002	1.7751e-001
13	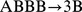	7.1993e-005	8.3475e-005	1.2110e-004
14	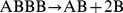	4.6617e-002	7.8662e-002	1.0304e-001
15	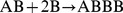	6.6738e-005	4.4724e-006	1.0953e-005

In column ***ExpT*** the reaction constants extracted by overlapping all datasets (20 observations). In column ***Gill*** we report the estimated reaction constants from 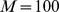 Gillespie simulations, fitting each single experiment and obtaining 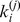, and then computing 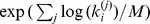, according to the lognormal distribution. In column ***Exp*** we report the same calculation for the 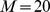 experiments.

We then run 100 Gillespie simulations using the previous values of the 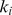, and we re-derived the effective value of the parameters by fitting the simulated data for each run with the deterministic model, as described in the previous Section. In this way we got a probability distribution of each parameter 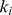.

**Figure 3:**
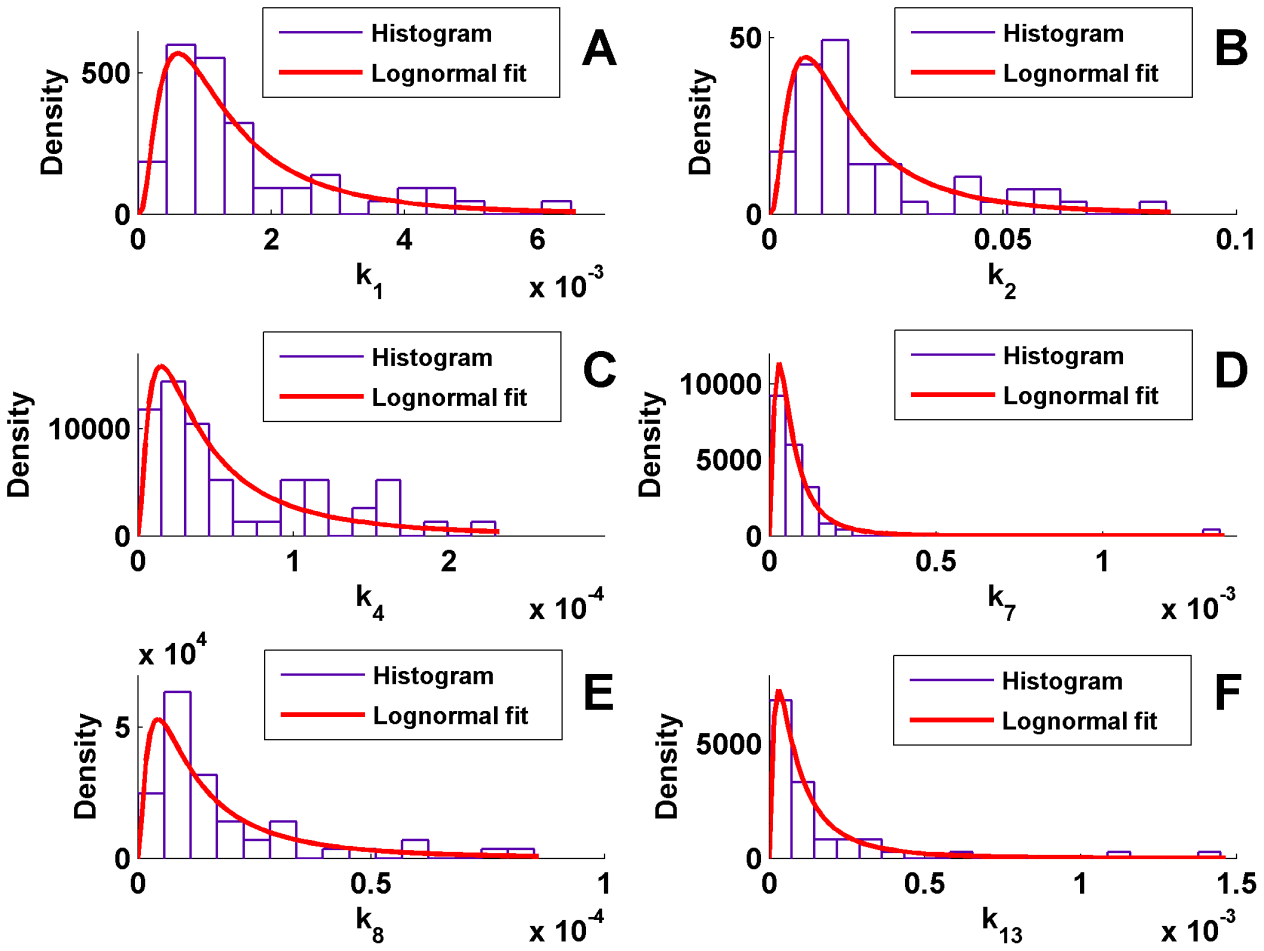
Distribution of the reaction coefficient. Coefficients 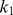 (A), 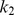 (B), 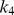 (C), 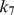 (D), 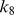 (E) and 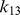 (F), obtained with our stochastic procedure; the best fit is achieved by means of Log-normal distribution comparing the likelihoods.

Some examples of the frequency distributions of parameter values 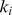 estimated from the stochastic model are shown in Fig. 3. The best fit of their distribution is given by a Log-normal distribution (we test also Exponential and Weibull), using the likelihood method. We proceeded in this way: we divided the 100 Gillespie simulations in two sets of 50. We then evaluated the parameters of the Log-normal distribution (average and variance) for the first set and used them in the likelihood test for the following 50 samples. The same was done dividing the 20 experimental samples in two sets of 10. The results are reported in Table 4

**Table 4:** The likelihood 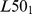 obtained from the first set of 50 stochastic simulation; the likelihood 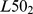 of the second set of 50 stochastic simulation evaluated using the distribution fit parameters achieved with the first set 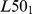; the likelihood 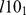 from the first set of 10 experiments; the likelihood 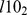 of the second set of 10 experiments evaluated using the distribution fit parameters achieved with the first set 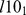; the Wilcoxon test 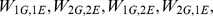 respectively, between the first stochastic set and the first experimental one, the second stochastic and the second experimental, the first stochastic and the second experimental, and finally the vice versa of the latter (0 accepted the zero hypothesis of the same distribution in which something belongs with significance level of 0.05, while if 1 the zero hypothesis is refused).

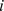	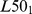	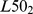	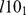	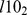	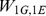	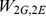	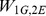	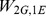
1	275.7832	258.1798	42.5066	38.8130	0	0	0	0
2	148.3391	129.0754	18.4377	19.8115	0	0	1	0
3	655.0960	662.2778	127.7790	128.7383	0	0	0	0
4	432.9189	424.9808	88.5726	86.0501	0	0	0	0
5	576.2967	575.9256	81.7484	66.1083	0	1	1	0
6	113.7160	105.6386	14.4388	6.4581	1	0	0	0
7	423.8807	410.9888	75.8147	78.1772	0	0	0	0
8	492.9463	500.2600	92.9226	55.4166	0	0	0	1
9	270.5161	255.1394	48.8032	53.9497	0	0	0	0
10	558.4369	569.1068	101.1988	92.7381	0	0	0	0
11	307.7186	281.8925	53.2616	53.7673	0	0	0	0
12	38.9892	44.7289	1.8639	−1.4582	1	0	0	1
13	392.4433	391.8237	72.1444	68.2362	0	0	0	1
14	72.6155	67.6900	5.3474	8.8448	0	0	0	0
15	484.5265	503.0466	82.1955	93.0230	0	0	0	1

The average values of the parameters obtained by means of the 100 Gillespie simulations are reported in Table 3, in the column labelled **Gill**. For comparisons, in the same table, column labelled **Exp**, we also report the same average for the 20 experiments. It can be noticed that the values obtained in these different ways are different, although being of the same order of magnitude.

**Figure 4:**
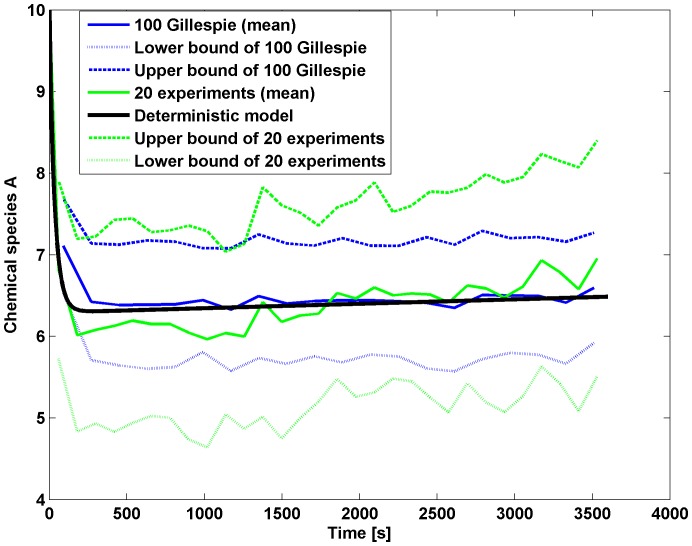
Fitting abundance of species A. Blue curve: average of 100 simulations of the stochastic model with Gillespie algorithm. Green curve: average of the experimental data. Red dotted and dash-dotted curves indicate the variance. The black line is the solution of the deterministic chemical model for the species 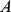.

**Figure 5:**
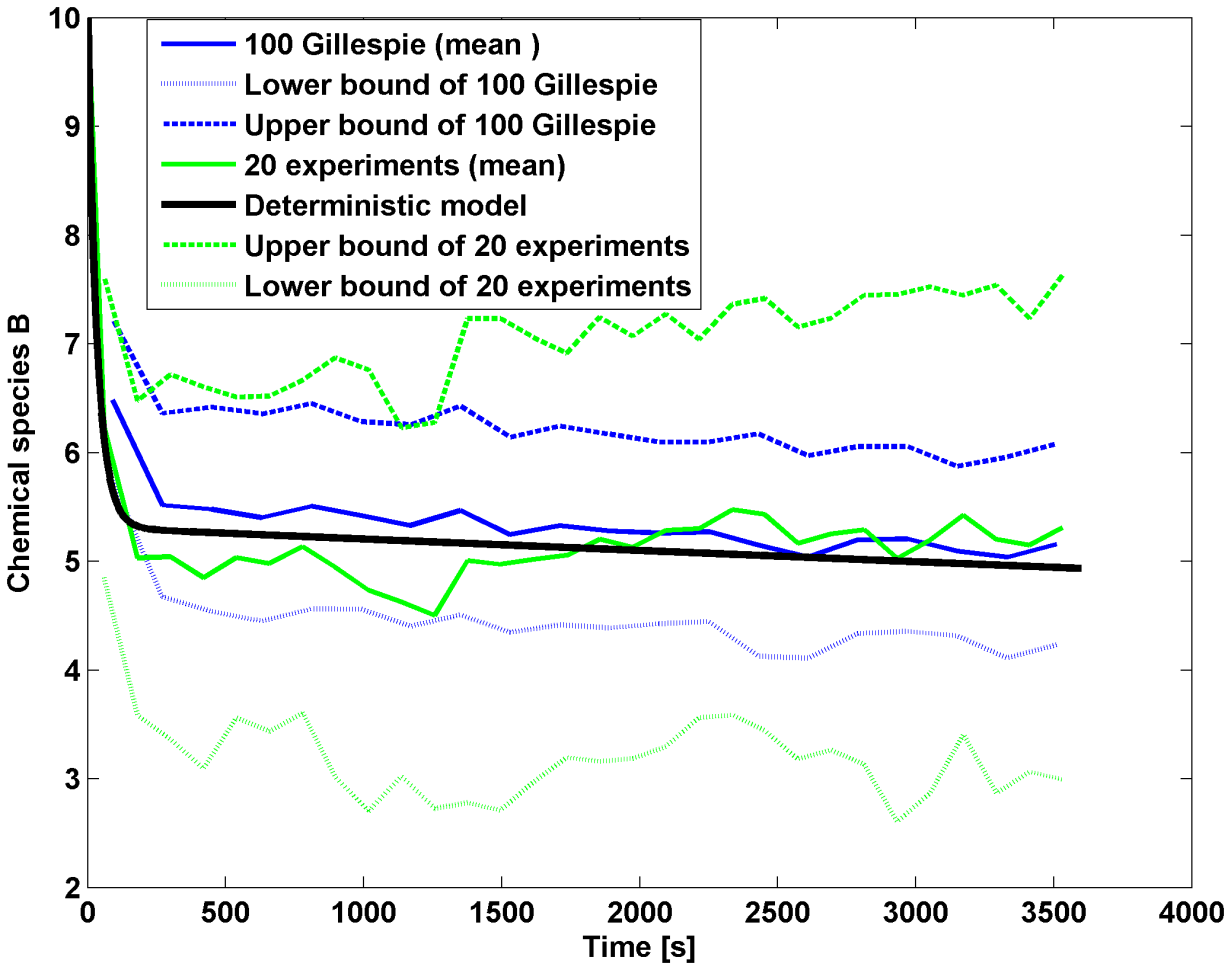
Fitting abundance of species B. Blue curve: average of 100 simulations of the stochastic model with Gillespie algorithm. Green curve: the average of the experimental data. Red dotted and dash-dotted curves indicated the variance. The black line is the solution of the deterministic chemical model for the species 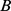.

**Figure 6:**
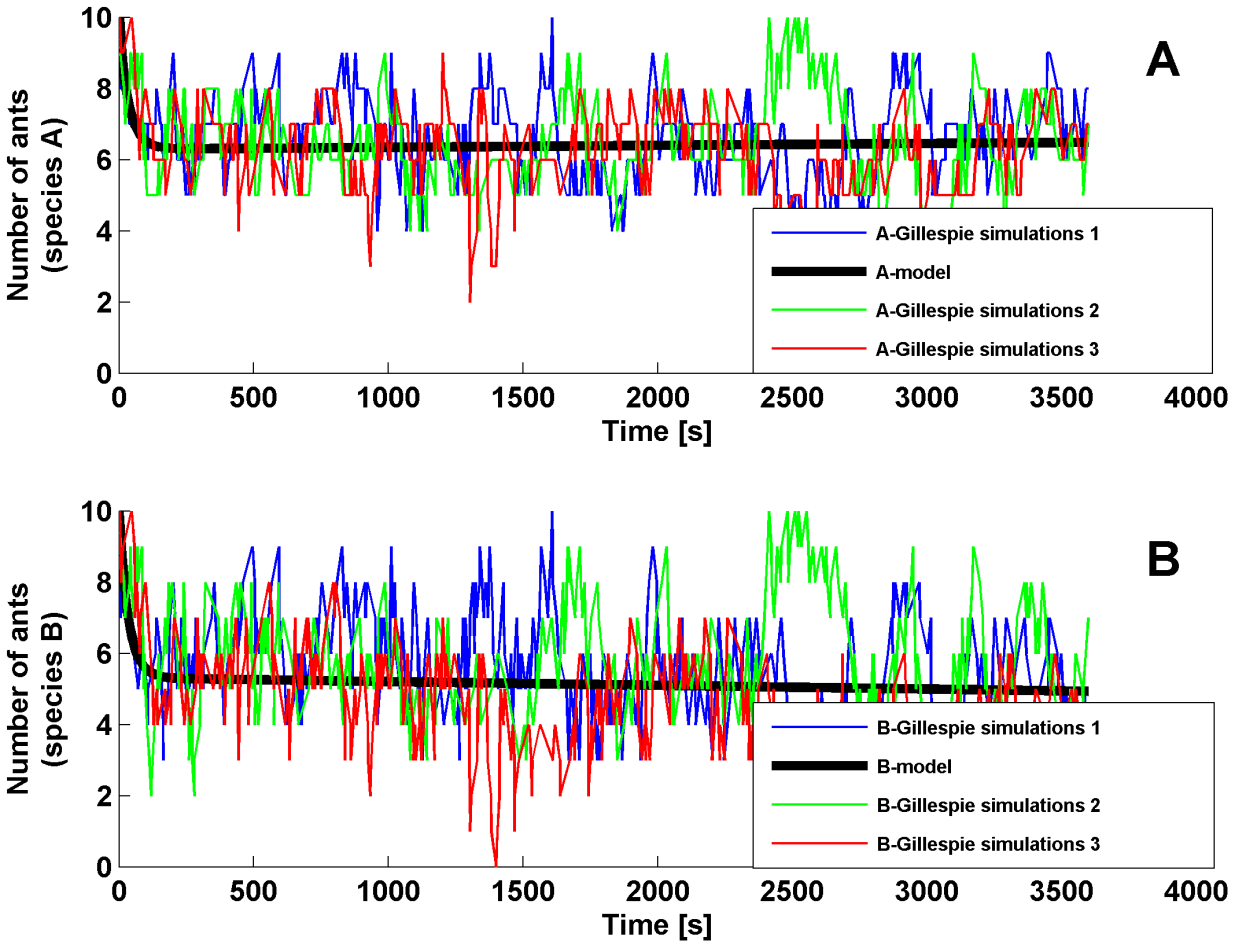
Stochastic time series. Temporal variation of the 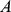 (A) and 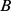 (B) chemical species, obtained from three simulations of the stochastic model. Predictions of the deterministic model are also shown (black line).

The average value of the observed chemical species 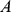 and 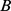, together with predicted values obtained from the deterministic model and the average of 100 stochastic simulations are reported in Fig. 4 and 5. Three examples of stochastic time series are shown in Fig. 6. Predictions from models well agree with experimental data and these are contained within the confidence bounds of the stochastic predictions. The corresponding time series for species 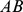, 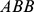 and 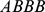, are shown in Figs. S7–S9.

In Table 4 we also show the likelihood achieved with our procedure and the results of Wilcoxon statistical test applied to the comparison of experimental and simulated reaction rates. For each reaction 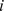 we get 50 values of the corresponding reaction rate 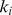 from the Gillespie simulations and compare it with 10 experimental values, in order to verify that they belong to the same distribution. The Wilcoxon statistical test is performed assigning the value 0 to the acceptance of the zero hypothesis (the two sets belong to the same distribution with a significance level of 0.05) and the value 1 to rejection of the zero hypothesis (see Table 4).

### Mortality experiments

The interaction experiments did not lasted long enough to allow an reliable estimation of the parameters related to casualties, Eqs. (2), (3), (5), (6), (9).

**Figure 7:**
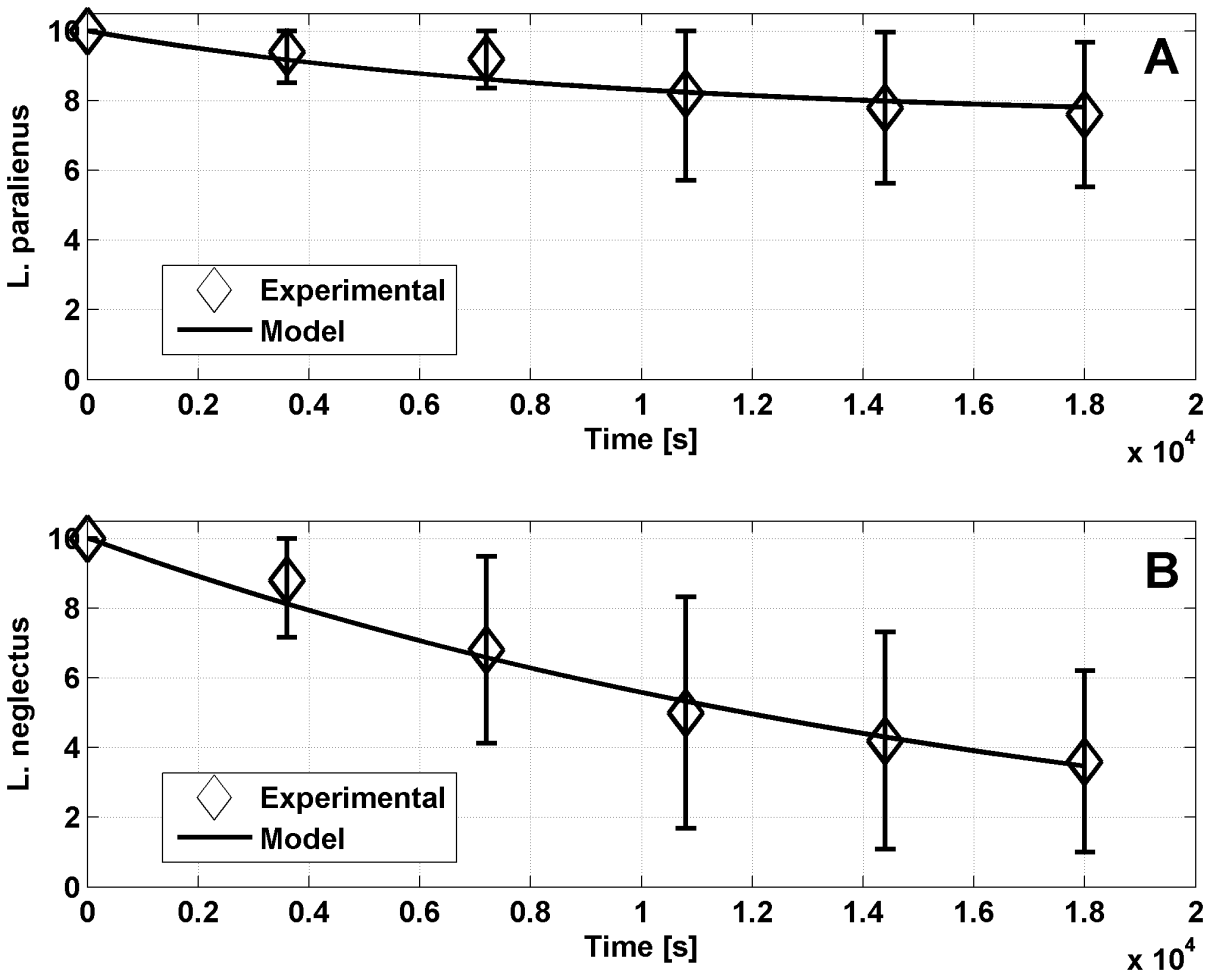
Mortality fitting. Calibration of the model with the 5-hour mortality experiments (black-line). The average of experimental data is indicated by diamonds: (A) species 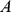 (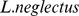), (B) species 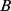 (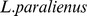).

We therefore performed longer experiments, denoted mortality, reported in Table 2. The estimated values of the parameters obtained when the deterministic model was fitted to mortality data (mean of 5 experiments with 10 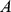 *vs*. 10 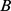) are reported in Table 5, column **ExpM**, while Fig. 7 shows the fitting curves for both species. In this calibration, only the mortality reaction coefficients were varied, while the others were kept constant, since the 5-hours experiments were recorded at a lower temporal resolution and it was not possible to keep track of all interactions.

**Table 5:** Values of estimated reaction constants (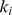) obtained using different sets of data.

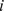	Reaction	ExpT	ExpM
1	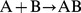	1.0063e-003	//
2	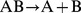	1.3130e-002	//
3	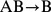	3.5892e-007	1.8305e-005
4	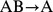	5.8290e-005	1.8074e-004
5	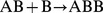	8.8645e-004	//
6	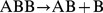	2.1387e-002	//
7	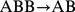	6.1787e-005	9.5488e-005
8	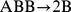	1.2312e-005	9.0222e-005
9	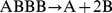	1.0528e-003	//
10	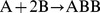	2.1974e-005	//
11	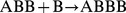	5.8482e-004	//
12	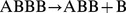	1.0246e-001	//
13	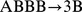	7.1993e-005	2.9154e-003
14	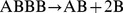	4.6617e-002	//
15	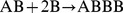	6.6738e-005	//

In column ***ExpT*** those from the *interaction* dataset (20 observations 10 vs. 10 for 1 hours), in column ***ExpM*** those from the mortality experimental dataset (5 observations 10 vs. 10 for 5 hours), limited to the reactions implying casualties.

### Supremacy phase diagram

We are now in the position of performing simulations of arbitrarily long battles with any number of opponents. Although the effective phase space of the autonomous system describing our model, Eq. 11 is five-dimensional, we have found that the projection of the trajectories on the plane 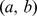 of the total number of 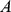 and 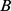 individuals (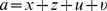, 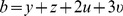) never intersect.

The results of these runs are shown in Fig. 1 as a phase plane portrait. A phase transition between two absorbing states, corresponding to zero-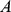 (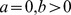) or zero-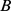 (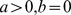) is evident. In other words, there is a critical value for the initial abundances of species 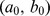 for which the final state is at the boundary separating the two absorbing states (*i.e.*, both species become extinct). The set of these critical values form a separatrix (red dashed line) which separates the initial states corresponding to the extinction of one or the other species. It is important to note that the phase separatrix is not a straight line, particularly when the size of the starting groups is small. This implies that the system is not simply represented by a superposition of small groups or, alternatively, that the large fighting groups have a non-negligible effect on the fate of the battle.

However, one can observe that the the separatrix becomes essentially linear when the abundances of the species are larger than about 10. Also the part of the diagram where the non-linearity of the trajectories is larger corresponds to the abundance of species less than 10. This linear behaviour over the 10-ants limit implies that a real battle, in which one can find many hundreds of individuals fighting, can be approximated by a superposition of local sub-battles, each of them composed by a small number of interacting individuals, of the order of ten individuals per species. This observation can be considered as a a *posteriori* justification for the small size of our experiments and constitutes an important element for experimental observations.

We also report in Fig. 1 the experimental results obtained by battles with different initial group sizes (see Table 2 for details). Each of the seven reported trajectories is the average of five different trials. The results shows a good agreement between the experimental data and the model output. The experimental trajectories essentially follow the flux lines of the deterministic model. Two of the trajectories cross the separatrix. This fact may be due to "shielding" of many 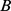 attacking a single 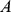, an effect that is not included in our model and that may have the effect of favouring the 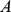 species, but that is important only for large 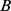/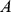 ratios near the separatrix, since well above it the final state is always the extinction of 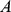, and well below it the final state is the extinction of 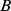. Indeed, all curves that start near the separatrix show an initial deviation favouring the 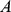 species.

## Discussion and Conclusions

In this paper we proposed a model based on a "chemical" description of aggressive interactions between opposing groups of cooperating animals. Our approach considers isolated individuals as atoms and fighting groups as molecules, and exploits the standard investigation tools of chemical reactions.

The model parameters were estimated using experimental observations of battles among a limited number (10) of individuals belonging to two species of ants (*L.paralienus* and *L.neglectus*). We developed both deterministic (mean-field) models and stochastic (Gillespie) ones.

The chemical approach proved to be simple enough to be adapted to a number of different cases, and was much more flexible and biologically meaningful than, for instance, Lanchester's one. One of its main strengths relies, in fact, in the ease with which realistic assumptions on the behaviour of the two groups can be explicitly coded and translated into observable characteristics. The latter point is of the utmost importance and cannot be overemphasized.

The main output of our model is the phase diagram shown in Fig. 1. First of all, although our model is five-dimensional, the projection of the trajectories in the 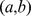 plane given by the total amount of 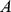 and 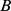 opponents shows no intersection, implying that these quantities are quite insensitive on the clusters that form and disaggregate during the battle. This point is important since it is much easier to estimate the total number of ants of each species than their aggregates. The fact that the trajectories and the separatrix are non-linear implies that there is a cooperative strategy in action. As illustrated below, it is given by the coordinated attack of multiple 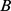 (*L.neglectus*) vs one 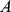 (*L.paralienus*). The comparison with experimental data is quite good.

The above scenario is confirmed by the analysis of the mortality rates. First of all *L. paralienus* (species 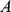), due to its size, has more possibilities to defeat *L. neglectus* (species 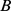) in a duel: the parameter 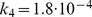 of reaction 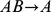 is ten times 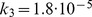, that of reaction 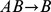 (the difference is even larger in the shorter interaction experiment, see Table 5).

By comparing 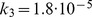 (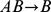), 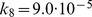 (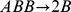) and 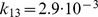 (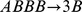) the cooperative strategy of *L. neglectus* becomes evident: by attacking *en masse* they can reach a higher killing ability; and 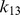 (three 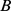 killing one 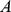) is larger than 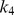 (one 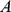 killing one 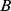). Clearly, this strategy depends on the possibility of having a larger number of opponents. Therefore, considering the coefficients of reactions that bring to death, we observe that the strategy of *L. neglectus* in 10 vs. 10 is not sufficient to defeat *L. paralienus*. The same consideration was also deduced with the stochastic model performing 1000 simulations with the Gillespie algorithm with the optimized parameters, in which we observe zero successes of the species 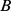, *i.e.*, all 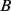 individuals die, while species 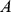 survives with a certain mortality. This is confirmed by the analysis in the phase plane of the deterministic model by varying the initial conditions, Fig. 1, that shows the importance of the initial ratio between the opponents. It also shows that the separatrix is well above the bisectrix (representing the advantage of 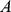 vs 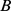 for low numbers of opponents).

We think that our approach is quite promising, although this present paper only represent a starting point. We are currently carrying out more detailed experiments, involving more ant species, in order to obtain a more careful quantitative estimation of the chemical parameters. Another direction is that of including the effects of fatigue. Our final goal is that of interpreting the chemical parameter in terms of a smaller number of factors (like aggressiveness, strength, cooperation, resistance, etc.) characteristic of each species.

## Supporting Information

Figure S1
**Chemical species** ***A*** **.** The chemical species 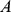 is shown as a function of time up to the steady state for three values of the initial conditions.(TIFF)

Figure S2
**Chemical species** ***B*** **.** The chemical species 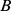 is shown as a function of time up to the steady state for three values of the initial conditions.(TIFF)

Figure S3
**Chemical species** ***AB*** **.** The abundance of the chemical species 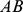 is shown as a function of time up to the steady state for three values of the initial conditions.(TIFF)

Figure S4
**Chemical species** ***ABB*** **.** The abundance of the chemical species 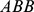 is shown as a function of time up to the steady state for three values of the initial conditions.(TIFF)

Figure S5
**Chemical species** ***ABBB*** **.** The chemical species 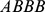 is shown as a function of time up to the steady state for three values of the initial conditions.(TIFF)

Figure S6 **Scheme.** Schematic representation of the proposed methodology followed to compare the deterministic model, expressed by means of a system of nonlinear differential equations, and the stochastic one.
(TIF)

Figure S7
**Experimental data and deterministic model for species** ***AB*** **.** The mean values of the chemical species 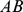 obtained from 20 experiments vs. the solution of the deterministic model by means of Simplex Flexible Algorithm(SFA).(TIFF)

Figure S8
**Experimental data and deterministic model for species** ***ABB*** **.** The mean values of the chemical species 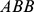 obtained from 20 experiments vs. the solution of the deterministic model by means of Simplex Flexible Algorithm(SFA).(TIFF)

Figure S9
**Experimental data and deterministic model for species** ***ABBB*** **.** The mean values of the chemical species 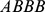 obtained from 20 experiments vs. the solution of the deterministic model by means of Simplex Flexible Algorithm(SFA).(TIFF)

File S1 **Supplementary material.** Integration of the differential equations. Gillespie implementation. Nonexistence of attractors.
(PDF)
