# Supplementary figures and images for: Modeling Warfare in Social Animals: A "Chemical" Approach
Source: PLoS One. 2014 Nov 4;9(11):e111310. doi: 10.1371/journal.pone.0111310 (PMC4219847; doi:10.1371/journal.pone.0111310)

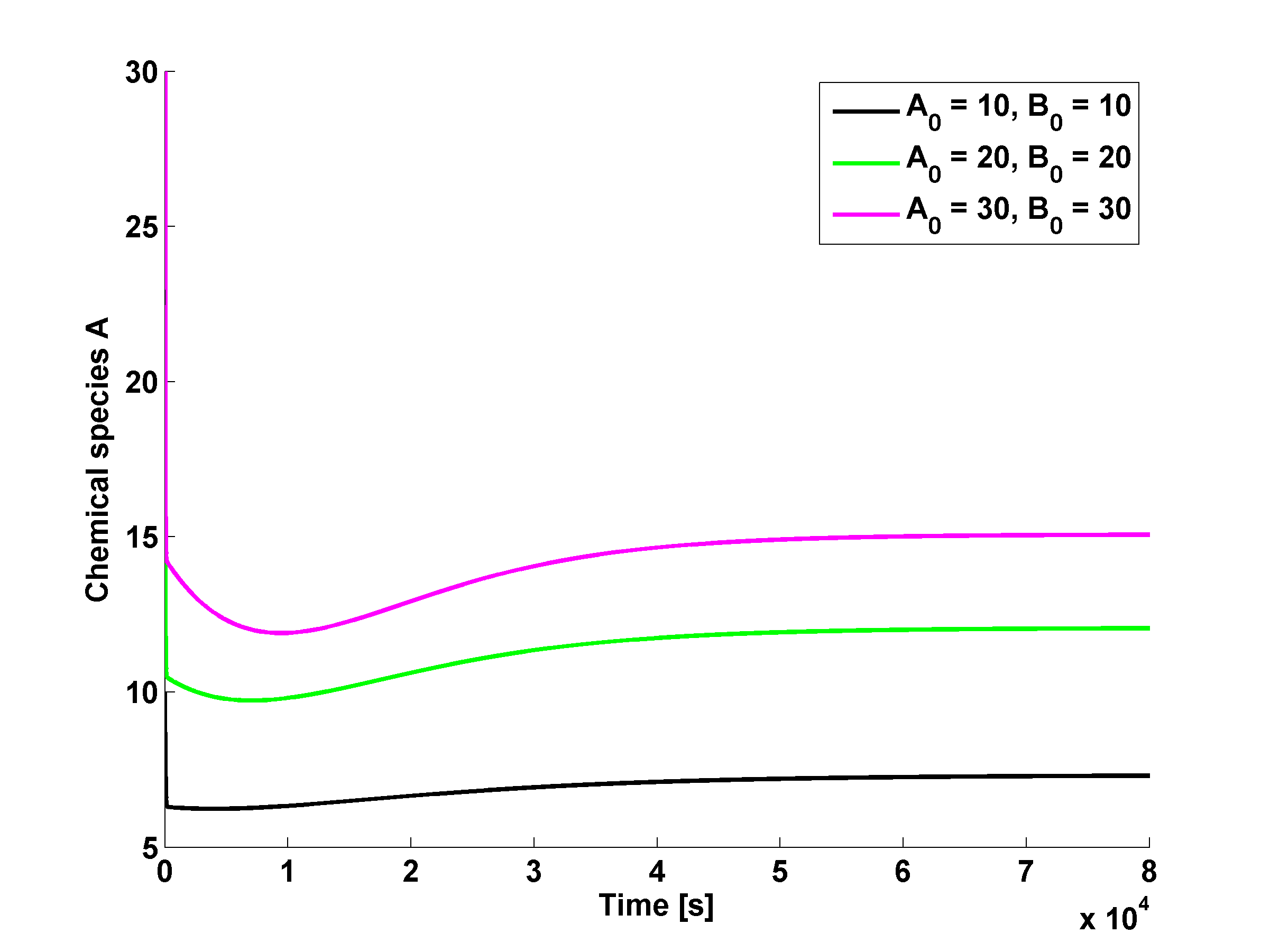

Supplement: Figure S1 — Chemical species A . The chemical species is shown as a function of time up to the steady state for three values of the initial conditions. (TIFF) [file pone.0111310.s001.tiff]

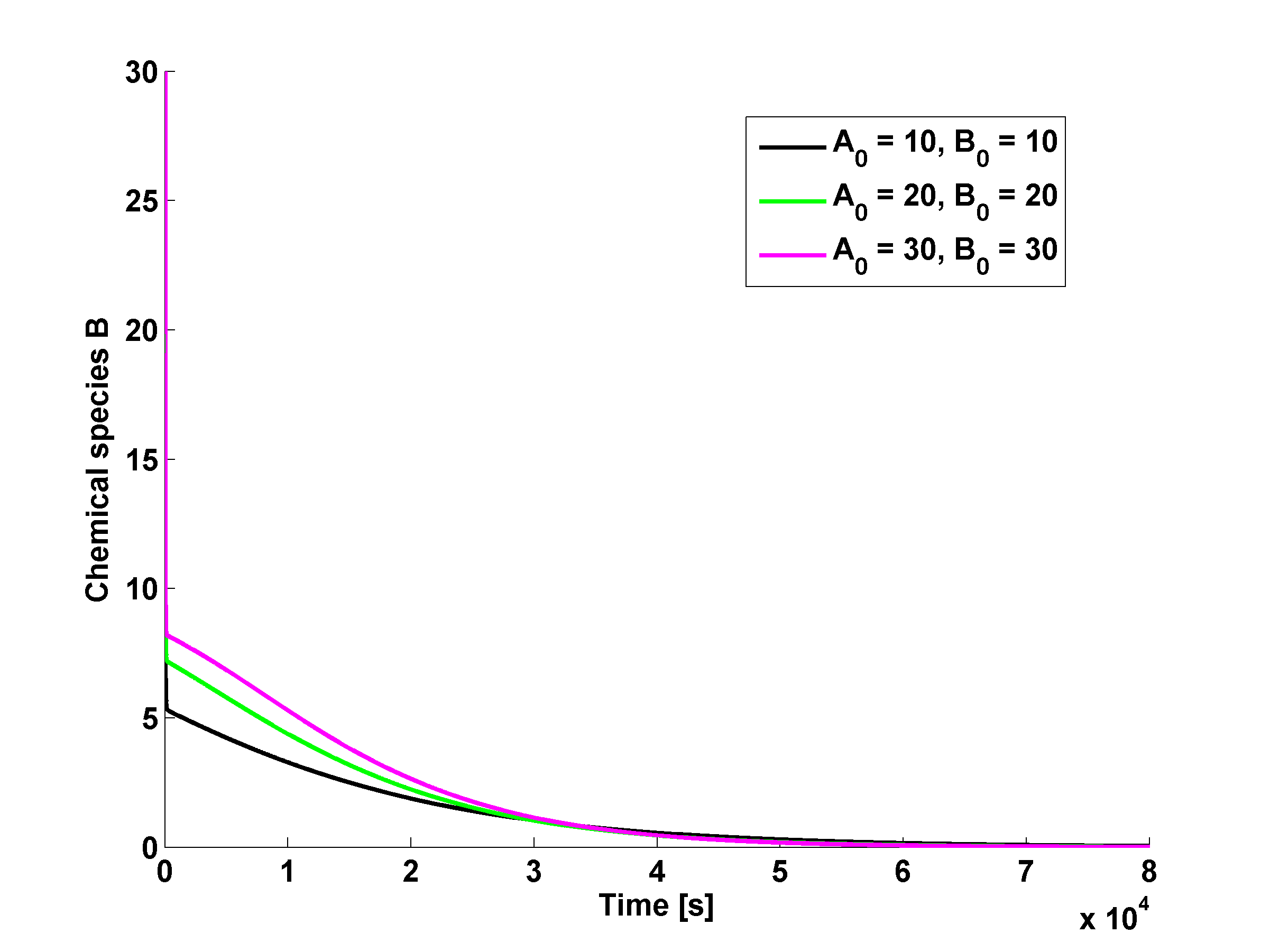

Supplement: Figure S2 — Chemical species B . The chemical species is shown as a function of time up to the steady state for three values of the initial conditions. (TIFF) [file pone.0111310.s002.tiff]

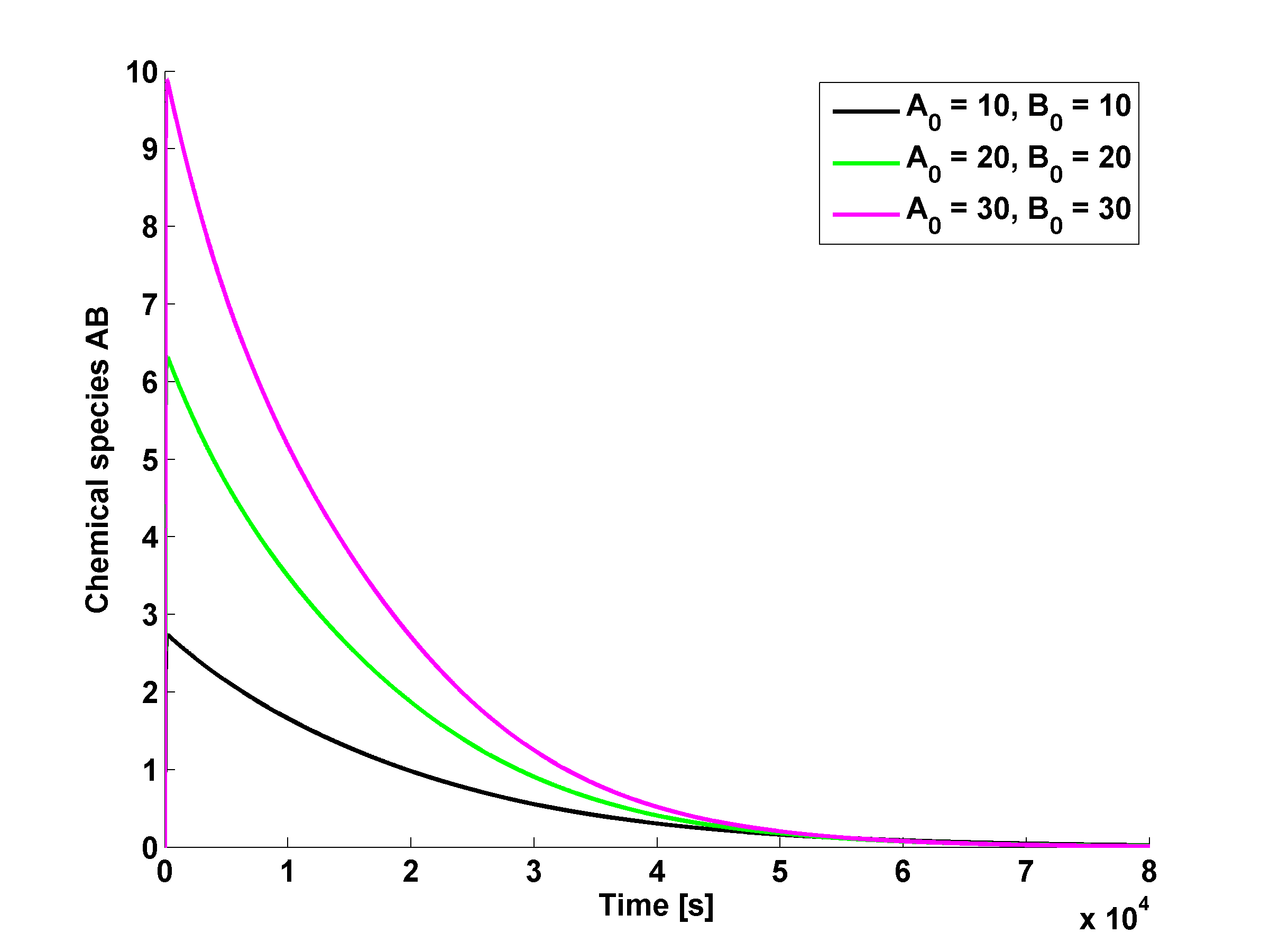

Supplement: Figure S3 — Chemical species AB . The abundance of the chemical species is shown as a function of time up to the steady state for three values of the initial conditions. (TIFF) [file pone.0111310.s003.tiff]

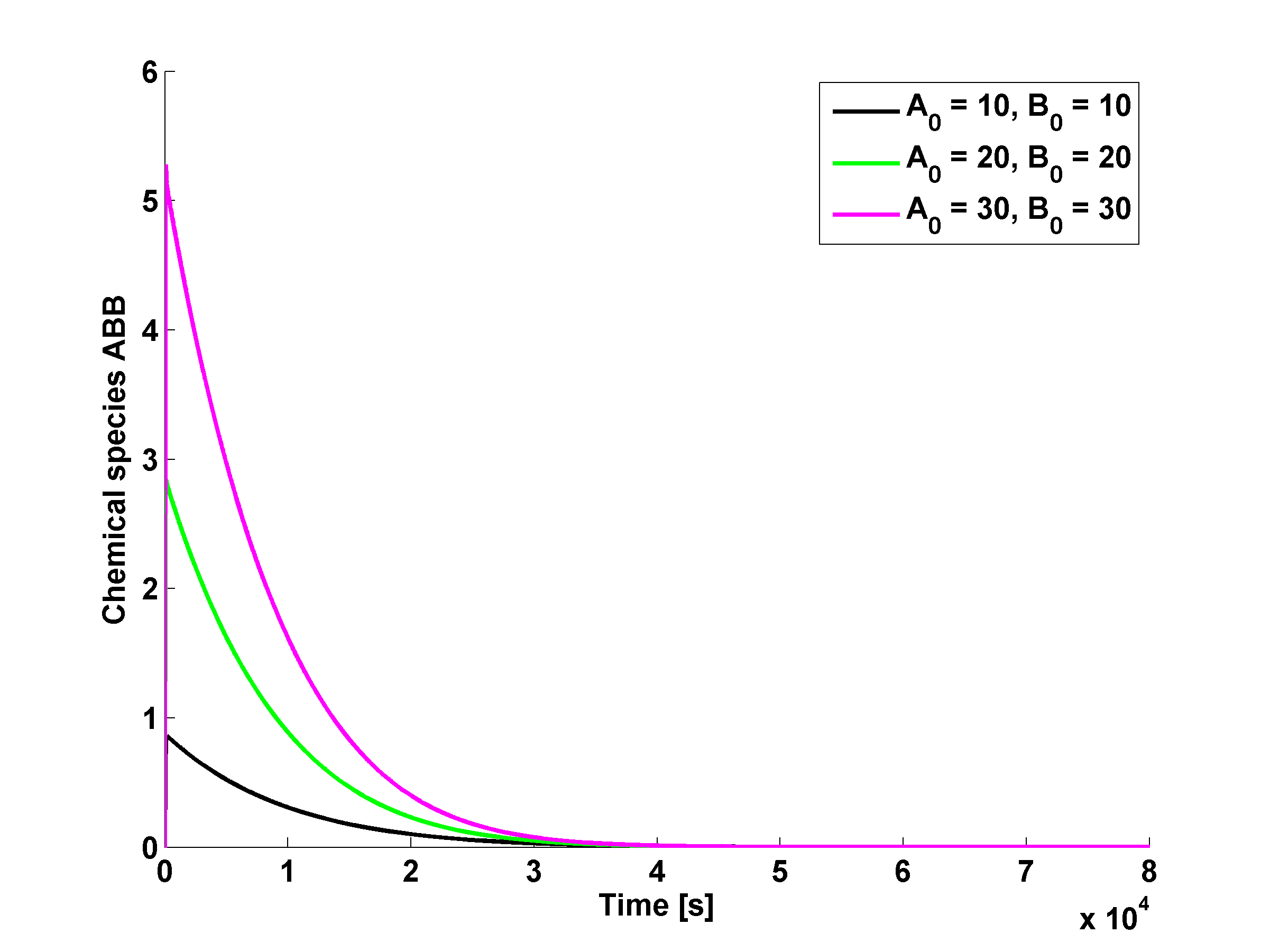

Supplement: Figure S4 — Chemical species ABB . The abundance of the chemical species is shown as a function of time up to the steady state for three values of the initial conditions. (TIFF) [file pone.0111310.s004.tiff]

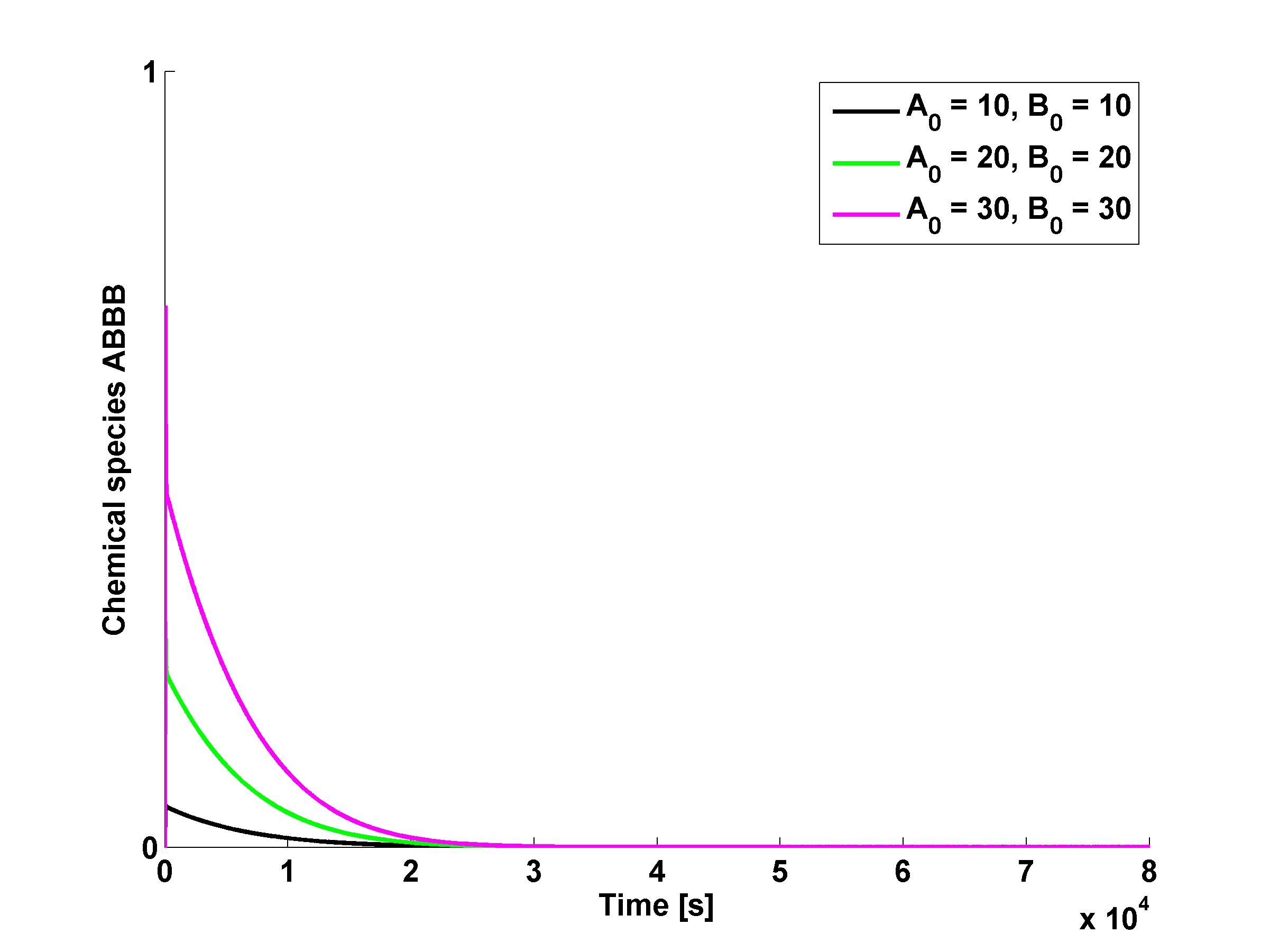

Supplement: Figure S5 — Chemical species ABBB . The chemical species is shown as a function of time up to the steady state for three values of the initial conditions. (TIFF) [file pone.0111310.s005.tiff]

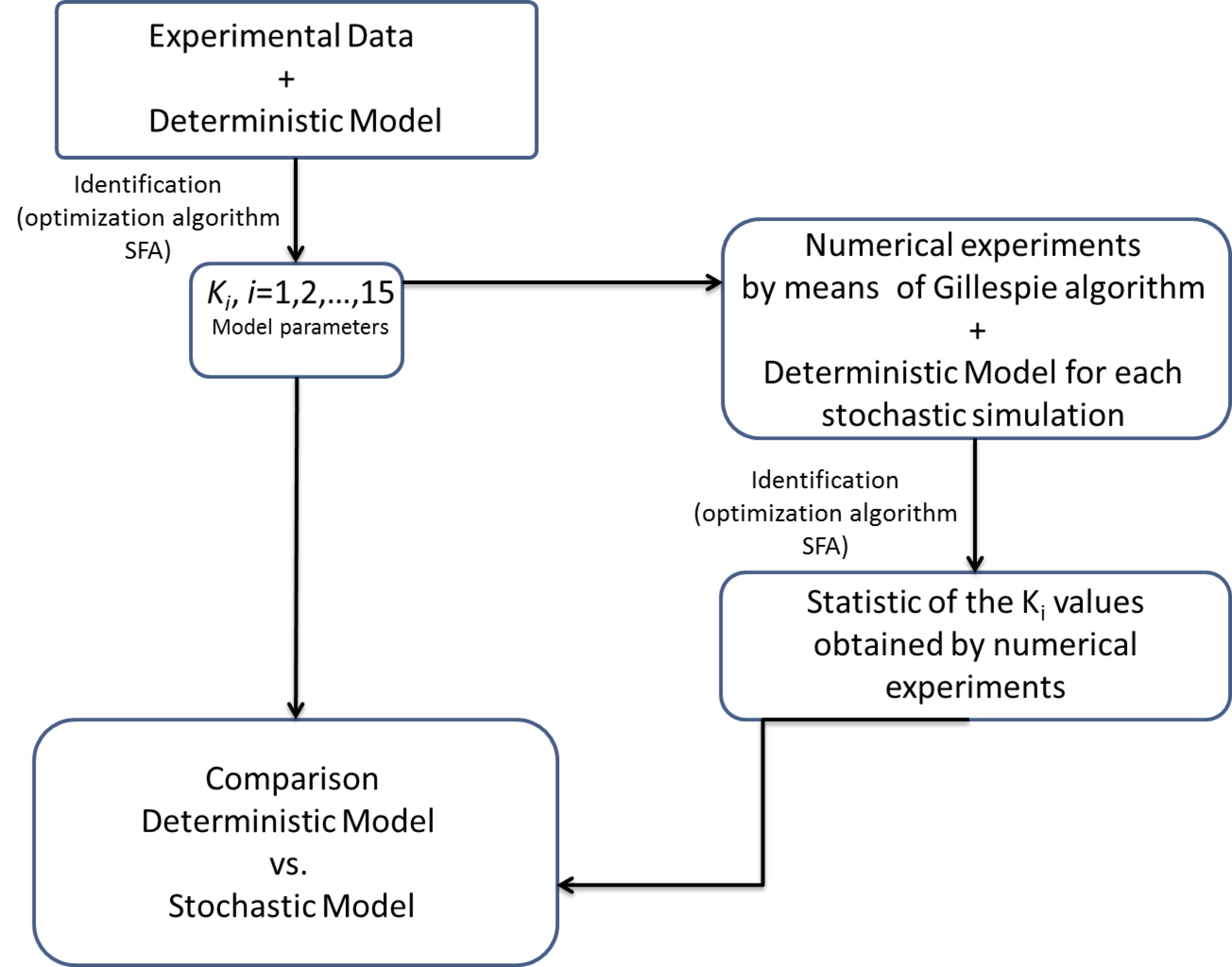

Supplement: Figure S6 — Scheme. Schematic representation of the proposed methodology followed to compare the deterministic model, expressed by means of a system of nonlinear differential equations, and the stochastic one. (TIF) [file pone.0111310.s006.tif]

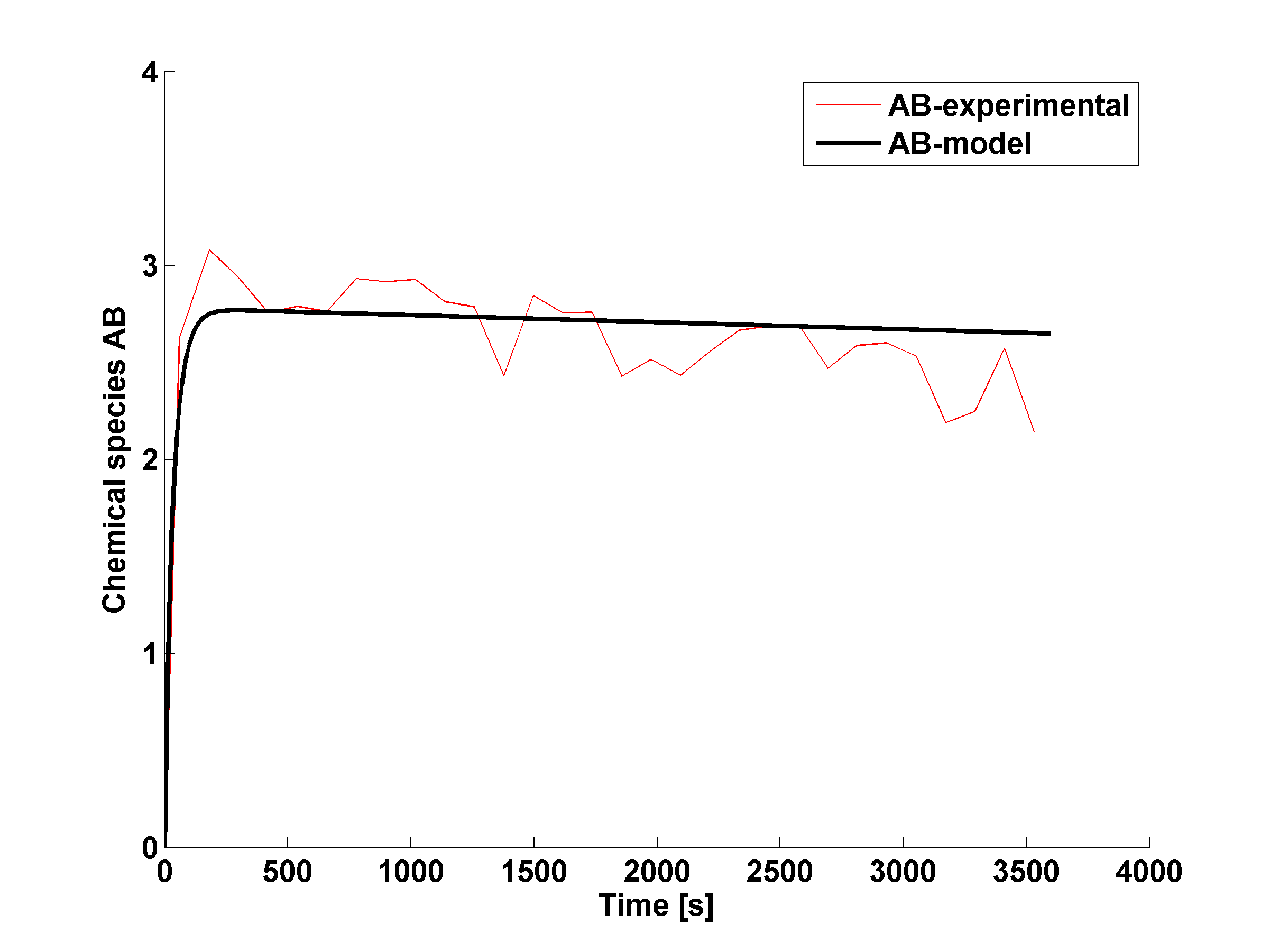

Supplement: Figure S7 — Experimental data and deterministic model for species AB . The mean values of the chemical species obtained from 20 experiments vs. the solution of the deterministic model by means of Simplex Flexible Algorithm(SFA). (TIFF) [file pone.0111310.s007.tiff]

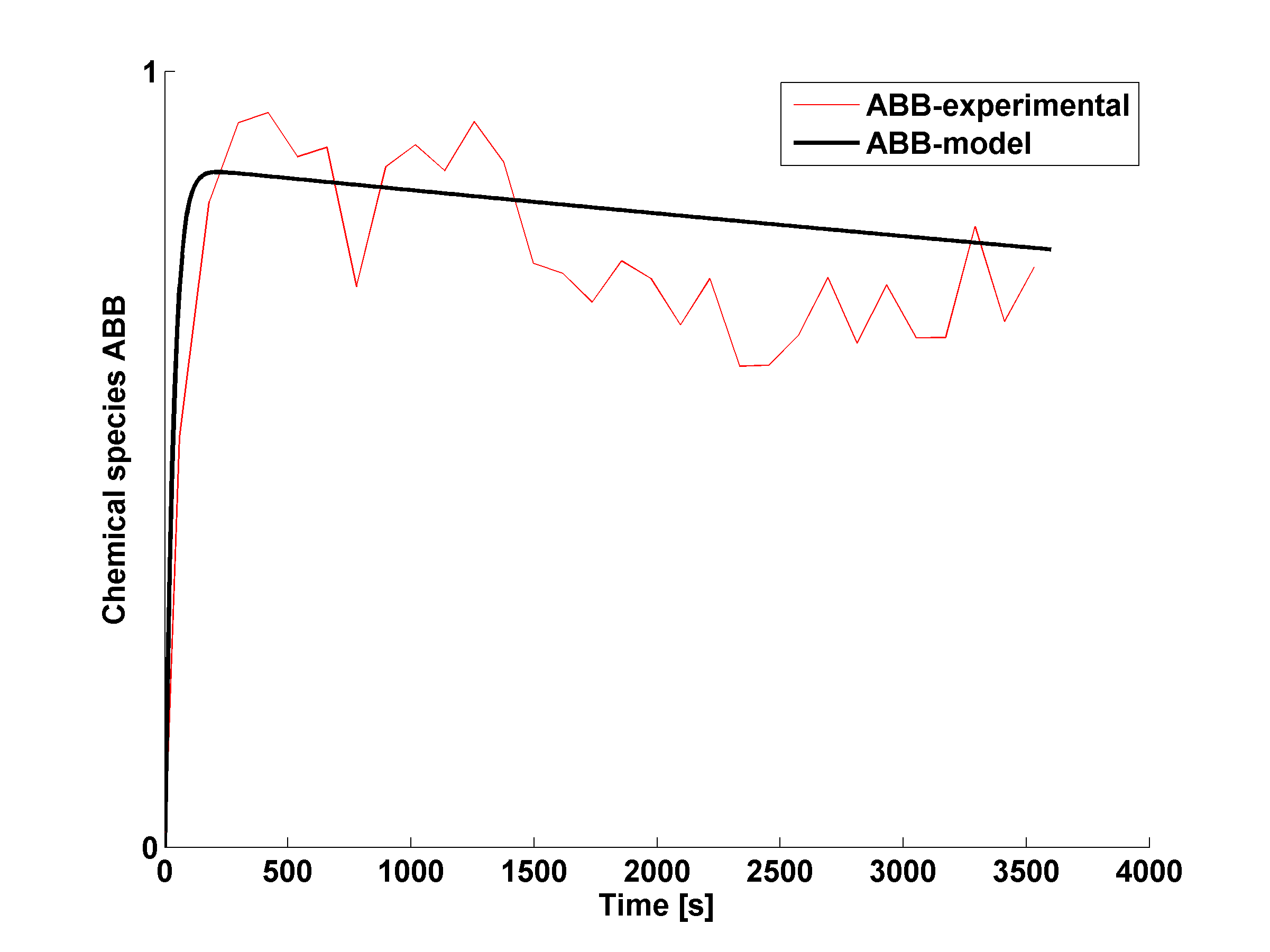

Supplement: Figure S8 — Experimental data and deterministic model for species ABB . The mean values of the chemical species obtained from 20 experiments vs. the solution of the deterministic model by means of Simplex Flexible Algorithm(SFA). (TIFF) [file pone.0111310.s008.tiff]

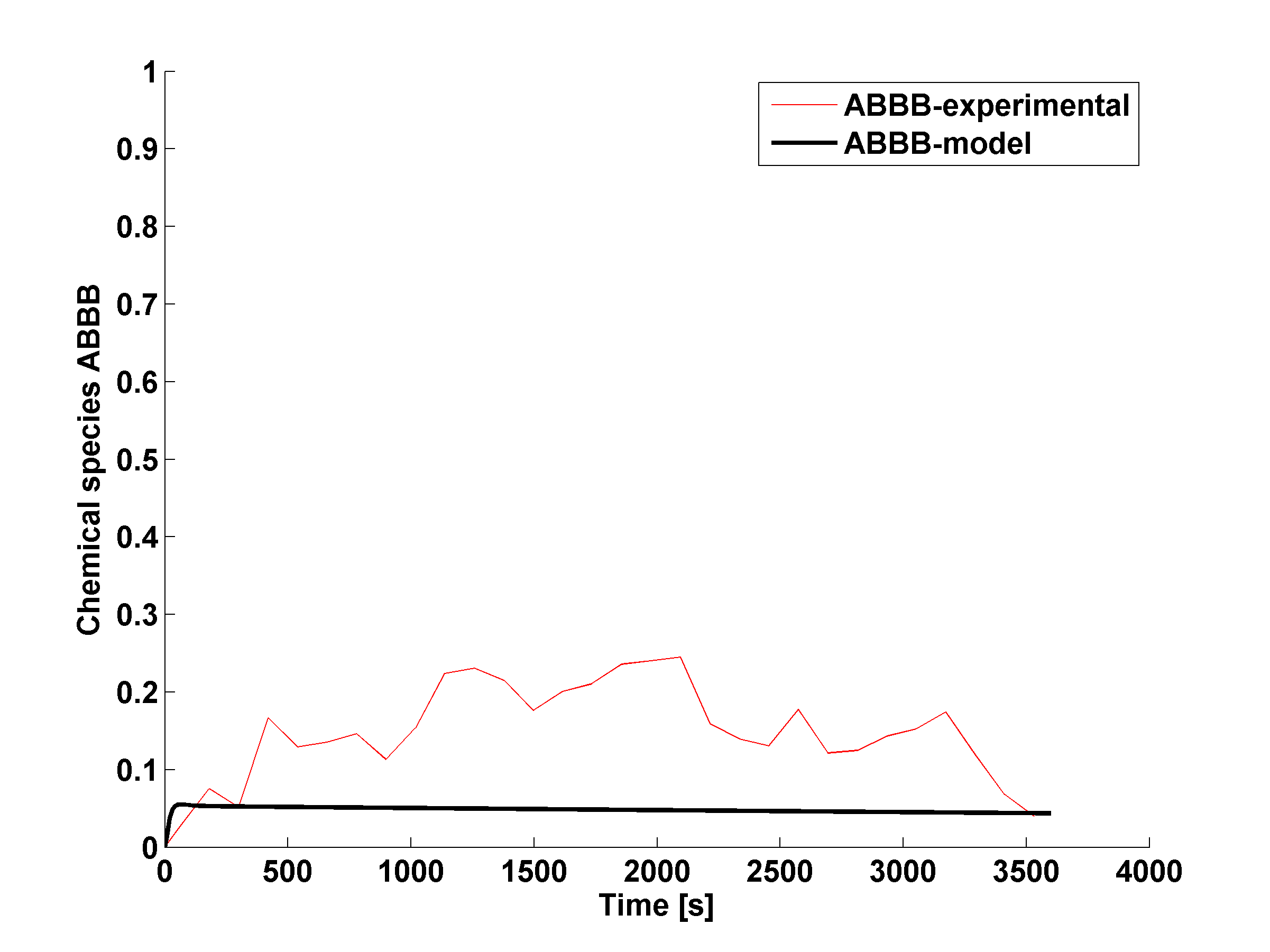

Supplement: Figure S9 — Experimental data and deterministic model for species ABBB . The mean values of the chemical species obtained from 20 experiments vs. the solution of the deterministic model by means of Simplex Flexible Algorithm(SFA). (TIFF) [file pone.0111310.s009.tiff]
