# Supplementary material for: Modeling Warfare in Social Animals: A "Chemical" Approach
Source: PLoS One. 2014 Nov 4;9(11):e111310. doi: 10.1371/journal.pone.0111310 (PMC4219847; doi:10.1371/journal.pone.0111310)
Supplement: File S1 — Supplementary material. Integration of the differential equations. Gillespie implementation. Nonexistence of attractors. (PDF) [file pone.0111310.s010.pdf]

Supplementary material for *Modeling warfare in social animals: a "chemical" approach.*

Alisa Santarlasci[1,3], Gianluca Martelloni[2,3],
Giacomo Santini[4], Franco Bagnoli[2,3,5,*]
1 Department of Information Engineering, University of Florence, Firenze, Italy.
2 Department Physics and Astronomy, University of Florence, Sesto Fiorentino (FI), Italy
3 Center for the Study of Complex Dynamics (CSDC), University of Florence, Sesto Fiorentino (FI), Italy.
4 Department of Biology, University of Florence, Sesto Fiorentino (FI), Italy.
5 National Institute of Nuclear Physics (INFN), sez. Firenze, Italy
∗ E-mail: franco.bagnoli@unifi.it

Integration of differential equations

In order to integrate this system of ordinary differential equations we use the numerical method of Cash and Karp [1] checking that the fourth and fifth order solutions provide the same results. This method is a member of the Runge-Kutta family of ordinary differential equation solvers.

Gillespie implementation

Table 1. The multiplicity factor g_i for the 15 reactions Eqs. (**??**)–(**??**).

i	g_i	i	g_i	i	g_i
1	xy	6	u	11	yu
2	z	7	u	12	v
3	z	8	u	13	v
4	z	9	u	14	v
5	yz	10	$\frac{1}{2}xy(y-1)$	15	$\frac{1}{2}zy(y-1)$

This method explicitly simulates each reaction, giving a stochastic formulation of chemical kinetics based on the theory of collisions. We have to compute, for each reaction, the number of ways in which it can be realized (multiplicity) and the probability density function of its occurrence during time (an exponential function), normalized over the sum of all reactions.

In our case, for each reaction i (out of the 15 of Eqs. 11) with reaction constant k_i, the propensity function is defined as $F_i = k_i g_i$, where g_i (the multiplicity factor) is the number of distinct ways in which the individual reactants of each species can be sampled in the population. The multiplicity factors g_i are reported in Table 1.

Considering a time interval Δt, the reaction probability density function of reaction i is $P(\Delta t, i) = P_1(\Delta t)P_2(j)$ (due to time homogeneity), with

$$
\begin{aligned}
P_1(\Delta t) =& F_0 \exp(-F_0 \Delta t),\\
P_2(i) =& \frac{F_i}{F_0},
\end{aligned}
$$

where $F_0 = \sum_{i=1}^{15} F_i$.

The two terms forming the probability density function are an exponential distribution of time reactions P_1 and the normalized propensity function P_2. The Gillespie algorithm can be implemented by choosing the next event time interval Δt as

$$\Delta t = -\frac{1}{F_0} \ln(r_1)$$

and the reaction i as the integer for which

$$\sum_{j=1}^{i-1} F_j < r_2 F_0 \leq \sum_{j=1}^{i} F_j,$$

where r_1 and r_2 are uniform random numbers between 0 and 1.

Nonexistence of attractors

As expected, this system does not exhibit fixed points, *i.e.*, the stationary solution ($\dot{x}_i = 0$) always depends on the initial conditions as can be seen in Fig. 1. The stationary condition is either $x \neq 0$ and $y = z = u = v = 0$ or $y \neq 0$ and $x = z = u = v = 0$. We can denote these two conditions as absorbing states for the dynamics. This is consistent with the biological point of view: the winning species and the the number of survivors depend on the initial number of individuals involved in the battle. Another view to illustrate the absence of the fixed point is to calculate directly the variations of the total number of A and B, denoted a and b. From Eqs. (11), we have

$$\begin{aligned} a &= x + z + u + v, \\ b &= y + z + 2u + 3v. \end{aligned} \tag{1}$$

After deriving and substituting Eqs. (11), we obtain

$$\begin{aligned} \dot{a} &= -k_3 z - k_8 u - k_{13} v, \\ \dot{b} &= -k_4 z - k_7 u, \end{aligned} \tag{2}$$

that simply show, as expected, the dependence of the total number of A and B from the mortality terms that depends only from the groups AB, ABB and $ABBB$. Since z, u and v are either positive or zero, the only stationary states $\dot{a} = \dot{b} = 0$ is given by the absence of any cluster ($z = u = v = 0$) and since with this condition

$$\begin{aligned} \dot{z} &= k_1 xy, \\ \dot{u} &= k_{10} xy^2, \\ \dot{v} &= 0, \end{aligned} \tag{3}$$

we have either $x = 0$ or $y = 0$ for the stationary state.

References

1. J. R. Cash, A. H. Karp. "A variable order Runge-Kutta method for initial value problems with rapidly varying right-hand sides", ACM Transactions on Mathematical Software **16**: 201-222 (1990). doi:10.1145/79505.79507
